# The Mutual Influence of Predominant Microbes in Sourdough Fermentation: Focusing on Flavor Formation and Gene Transcription

**DOI:** 10.3390/foods11152373

**Published:** 2022-08-08

**Authors:** Tongjie Liu, Yixin Shi, Yang Li, Huaxi Yi, Pimin Gong, Kai Lin, Zhe Zhang, Lanwei Zhang

**Affiliations:** 1 College of Food Science and Engineering, Ocean University of China, Qingdao 266003, China; 2 College of Food Science and Engineering, Qingdao Agricultural University, Qingdao 266109, China

**Keywords:** sourdough, volatile compounds, flavor, RNA sequencing, interplay

## Abstract

The interplay between microorganisms generally plays a vital role in food fermentation. In this study, the mutual influence of *Saccharomyces cerevisiae* and *Fructilactobacillus sanfranciscensis,* the two predominant microbes in the sourdough ecosystem, were investigated in situ during fermentation. Doughs fermented with *S. cerevisiae*, *F. sanfranciscensis,* or their combination were compared regarding acid production, microbial density, and volatiles. Furthermore, in situ gene expressions were investigated using RNA-sequencing. The results showed that the presence of *S. cerevisiae* had no visible influence on *F. sanfranciscensis*, whereas *F. sanfranciscensis* facilitated the growth of *S. cerevisiae* but affected its volatile production since metabolites such as 3-methyl-1-butanol decreased. The RNA-sequencing demonstrated that *S. cerevisiae* significantly changed the gene transcripts implicated in amino acid metabolism in *F. sanfranciscensis* and may stimulate its growth suggested by the enrichment of the KEGG pathway of peptidoglycan biosynthesis.

## 1. Introduction

Sourdough, a fermented mixture of water and flour, has traditionally been used as a starter culture in cereal-based fermentation worldwide [1]. Sourdough fermentation positively affects the final product, e.g., sourdough bread, in terms of flavor, texture, nutrition, and shelf life, due to the metabolic activities of the inherent microbiota [2]. A myriad of research has revealed that sourdough microbiota mainly consists of lactic acid bacteria (LAB) and yeast, and particularly, *Fructilactobacillus sanfranciscensis* and *Saccharomyces cerevisiae* are the most representative bacteria and yeast species, respectively [3,4,5]. The collaboration of LAB and yeast facilitates sourdough fermentation, the former mainly performing acidification, the later leavening dough, and both contributing to flavor formation [3]. It should be emphasized that their metabolic activities are not independent. Generally, they are mutually influenced and exhibit interactions to remain a stable association in this specific niche [6]. For instance, the collaboration between *F. sanfranciscensis* and maltose-negative yeasts, e.g., *Kazachstania exigua*, in the utilization of maltose and proteins during sourdough fermentation is a well-known example of mutualistic interaction [7,8]. Furthermore, it has been recently reported that *S. cerevisiae* could produce growth factors stimulatory to LAB [9].

Most traditional fermented foods are produced by a mixture of microbes, and the interplay between them is significant to their performance in food fermentation, thereby influencing the quality of end-product [10]. The flavor is one of the essential attributes of any food, determining its acceptance by consumers [11]. It has been reported that the interactions between microorganisms play a crucial role in the flavor formation of fermented products by influencing the generation of volatile metabolites, such as in the cases of cheese [12], wine [13], and Chinese liquor [14]. As the two main microbial species in sourdough, the interactions between *F. sanfranciscensis* and *S. cerevisiae* are supposed to be an important factor influencing the flavor of bread via changing the relative yields of volatile metabolites [15]. For instance, it has been found that in sourdough fermentation, the presence of *S. cerevisiae* favored acetic acid production. In contrast, ethanol production was affected negatively by the presence of LAB, including *F. sanfranciscensis* [16]. Noticeably, acetic acid and ethanol are among the most cited flavor compounds in bread [17].

Currently, omics approaches have been widely employed to elucidate microbial structures and decipher underlying metabolic activities in various fermentation ecosystems, including sourdough [18]. However, the transcriptomics study of sourdough fermentation is still lacking, and the profiles of involved genes are unclear. In addition, though previous studies have investigated the metabolic interactions between yeast and LAB isolated from sourdough in terms of substrate utilization, they were mainly performed in defined media, scarcely in situ. Therefore, this study investigated in situ interactions between *F. sanfranciscensis* and *S. cerevisiae* during sourdough fermentation regarding dough acidification, cell proliferation, and flavor formation. Furthermore, in situ gene expressions of the two microorganisms were deciphered using RNA sequencing to deeply elucidate the interactions during sourdough fermentation at the gene level.

## 2. Materials and Methods

### 2.1. Strains and Cultural Media

Two strains, *Saccharomyces cerevisiae* 9Y3 and *Fructilactobacillus sanfranciscensis* LS1 were isolated from traditional sourdough in our previous work [19,20] and were used singly or in combination to initiate dough fermentation. The sourdough bacteria (SDB) broth [21] was used for the cultivation of *F. sanfranciscensis* LS1, while *S. cerevisiae* 9Y3 was cultured in yeast peptone dextrose medium (YPD, 1% yeast extract, 2% peptone, 2% glucose, *m*/*v*). The strains were stored at −80 °C before use.

### 2.2. Wheat Flour and Sourdough Manufacturing

The wheat flour used in this study and its properties were described in our previous study [22]. For the sourdough/dough making, the strains were activated twice in the corresponding broth at 30 °C for 24 h; then, they were incubated for 48 h at 30 °C with an inoculum size of 1% (*v*/*v*). The cells were harvested by centrifugation (3000× *g* for 15 min at 4 °C) and washed twice. Finally, they were resuspended in sterile distilled water to a final concentration of 9 log cfu/mL (for *F. sanfranciscensis* LS1) and 8 log cfu/mL (for *S. cerevisiae* 9Y3). They were then used as either singular or combination starter cultures to make sourdough/dough. Briefly, fifteen grams of wheat flour, the starter suspension, and sterile distilled water (the latter two were 15 g in total, and the amount of sterile distilled water was adjusted according to the quantity of starter suspension added) were thoroughly mixed under aseptic conditions as previously described [22] and incubated at 30 °C for 12 h. The final concentrations of *F. sanfranciscensis* LS1 and *S. cerevisiae* 9Y3 in sourdough were 7 log cfu/g and 5 log cfu/g, respectively. The flour was the only ingredient that was not sterilized in the sourdough-making process. Unsterilized flour was chosen in this study to elucidate the interactions between *F. sanfranciscensis* and *S. cerevisiae* under a nearly natural condition, without destroying the inherent microbes and affecting the nutrients in the flour by the sterilization process. The experiment was repeated at least three times.

### 2.3. Determination of the pH and Microbial Loads

The pH determination and enumeration of LAB and yeast were performed as previously described with minor modifications [19,20]. Briefly, ten grams of each sample was added to 100 mL of distilled water and homogenized manually using a glass rod until the sample was thoroughly suspended. The pH was determined using a pH meter (PB-10, Sartorius, Germany). As for the enumeration, five grams of each sourdough were suspended in 45 mL sterile saline and decimally diluted [20]. *F. sanfranciscensis* was cultured on the SDB agar containing 0.1 g/L of cycloheximide (Sigma-Aldrich, St. Louis, MO, USA) and incubated anaerobically at 30 °C for 48 h. *S. cerevisiae* was plated on YPD agar supplemented with 0.1 g/L of chloramphenicol (Sigma-Aldrich, St. Louis, MO, USA) and incubated aerobically at 30 °C for 48 h.

### 2.4. RNA Extraction and Sequencing Library Preparation

The total RNA was directly extracted from the sourdoughs after the 12 h fermentation according to the method previously described [22]. Briefly, ten grams of sourdough was thoroughly mixed with 10 mL of sterile ultrapure water aseptically as described above, and the first centrifugation (1000× *g*, 5 min, 4 °C) followed. The collected supernatant was subjected to second centrifugation (5000× *g*, 15 min, 4 °C), and the obtained precipitate was used for subsequent RNA extraction. The total RNA was extracted using the TRIzol^TM^ Reagent (Invitrogen, CA, USA) and purified with the RNeasy^®^ Mini kit (Qiagen, Hilden, German) according to manufacturers’ protocols (TRIzol™ Reagent User Guide-Pub. No. MAN0001271 and RNeasy^®^ Mini Handbook). The purity and concentration of the extracted RNA were checked by agarose gel electrophoresis and measured using Nanodrop 2000 (Thermo Fisher Scientific, Wilmington, DE, USA). Then, the messenger RNA (mRNA) of *F. sanfranciscensis* and *S. cerevisiae* was enriched by removing ribosomal RNA (rRNA) using the Ribo-Zero rRNA Removal Kit (Gram-Positive Bacteria) and Ribo-Zero Gold rRNA (Yeast), respectively, provided by Illumina (San Diego, CA, USA), following manufacturer’s protocols. Finally, the sequencing libraries were prepared with the enriched mRNA following the Illumina TruSeq^®^ Stranded mRNA Sample Preparation Guide, which mainly included purification and fragmentation of mRNA, synthesis of the first and second strand complementary DNA (cDNA), adenylation of 3′ ends, ligation of adapters, enrichment of DNA fragments, library validation, and normalization and pooling of the libraries.

### 2.5. Library Sequencing and Data Treatment

The constructed libraries, loaded into an cartridge (MiSeq^®^ v2 Reagent Kit) provided by Illumina (San Diego, CA, USA), were sequenced using a 2 × 150 bp paired-end method on an Illumina MiSeq platform. The generated sequences were subjected to adapter trimming and removal of low-quality bases (Q score < 30) and short sequences (<20 bp) by the real-time analysis software built in the Miseq platform. Specifically, the adapters were trimmed using the software cutadapt version 1.14, and the reads quality was checked using the software FastQC version 0.11.5. Then the filtered raw data were analyzed on the Majorbio Cloud platform (https://cloud.majorbio.com/, accessed on 23 May 2019), an online platform for high-throughput omics data analysis [23]. The mapping to a reference genome and the annotation of transcripts was conducted based on the sequenced strains *F. sanfranciscensis* TMW 1.1304 and *S. cerevisiae* S288C. The gene expression level of the annotated transcripts was normalized using the transcripts per million (TPM) method [24]. An FDR-adjusted *p* value of <0.05 and a fold change of ≥2 or ≤0.5 was used as the criterion to select differentially expressed genes (DEGs). Functional annotation and enrichment analysis were applied to the DEGs with databases including gene ontology (GO) and the Kyoto encyclopedia of genes and genomes (KEGG).

### 2.6. Determination of Volatile Compounds in the Sourdough

The volatile compounds in the sourdoughs fermented for 12 h were determined as previously described [22]. Briefly, headspace solid-phase microextraction (HS-SPME) was employed to extract the volatile compounds. The fiber 75 μm carboxen/polydimethylsiloxane (CAR/PDMS) purchased from Supelco (Bellefonte, PA, USA) was used [25]. Three grams of the sourdough sample and one gram of NaCl were loaded into a 20 mL headspace vial sealed with screw caps, followed by equilibration of 15 min at 60 °C. Then, the volatile compounds in the headspace were absorbed with the fiber in a 30 min extraction. The volatile compounds were desorbed and identified using gas chromatography-mass spectrometry (GC-MS). The GC-MS conditions and the methods used for compound identification were the same as in our previous study [20]. The semiquantitative analysis of each volatile compound was performed by integrating a specific ion [26], and the integral areas of the identified compounds were employed to form a dataset for multivariate analysis.

### 2.7. Standards

Standards were purchased for volatile compound identification. 2-Pentylfuran and trans-3-octen-2-one were provided by Sigma Aldrich (St. Louis, MO, USA). Other compounds, including hexanal, benzaldehyde, octanal, nonanal, heptanal, 3-methylbutanal, propanol, pentanol, hexanol, ethanol, 1-octen-3-ol, 3-methyl-1-butanol, 2-methylpropanol, phenethyl alcohol, acetoin, 6-methyl-5-hepten-2-one, 2,3-butanedione, acetic acid, octanoic acid, pentanoic acid, hexanoic acid, γ-undecalactone, ethyl acetate, hexyl acetate, were provided by Macklin (Shanghai, China).

### 2.8. Statistical Analysis

The dataset mentioned above was subjected to a Pearson pretreatment followed by a principal component analysis (PCA) using the software XLSTAT (version 2018.5) to compare the sourdoughs fermented with different starters in terms of their volatile profiles. One-way analysis of variance (ANOVA) and multiple comparisons were performed on the integral areas of the identified compounds using SPSS software (version 23).

## 3. Results and Discussion

### 3.1. Acidity and Microbial Loads of the Fermented Dough

**Figure 1:**
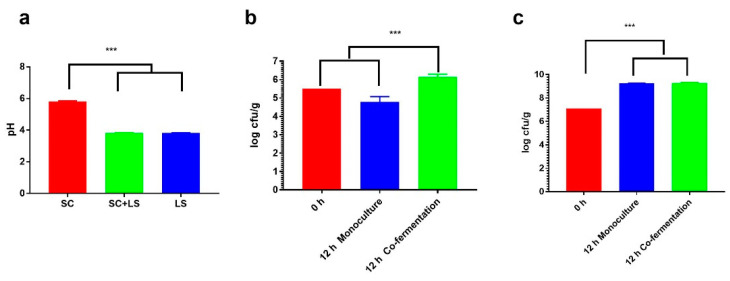
The pH values (**a**) and changes in microbial population of *S. cerevisiae* 9Y3 (**b**) and *F. sanfranciscensis* LS1 (**c**) in sourdoughs under monoculture and co-fermentation conditions. (*** *p* < 0.001), SC: *S. cerevisiae* 9Y3; LS: *F. sanfranciscensis* LS1.

The pH values of the dough fermented by *F. sanfranciscensis* LS1 (LS), *S. cerevisiae* 9Y3 (SC) and their combination (LS + SC) for 12 h were determined. As shown in Figure 1a, compared to the dough fermented by *S. cerevisiae*, the pH value of the co-fermented dough was significantly decreased (3.80 ± 0.00 vs. 5.80 ± 0.02, *p* < 0.001); on the contrary, the pH value of dough fermented by *F. sanfranciscensis* did not show a difference from that of the co-fermented dough (3.80 ± 0.00 vs. 3.80 ± 0.00), indicating that the presence of *S. cerevisiae* did not affect the acid production capacity of *F. sanfranciscensis*, which was in accordance with the previous report [16]. As for the microbial loads, the final bacteria concentrations in the single starter fermentation and co-fermentation were almost the same for *F. sanfranciscensis*, which was boosted by two orders of magnitude than the initial inoculum level (Figure 1b).

This demonstrated that the presence of *S. cerevisiae* did not negatively affect the proliferation of *F. sanfranciscensis*, validating the results of previous studies [16].

**Figure 2:**
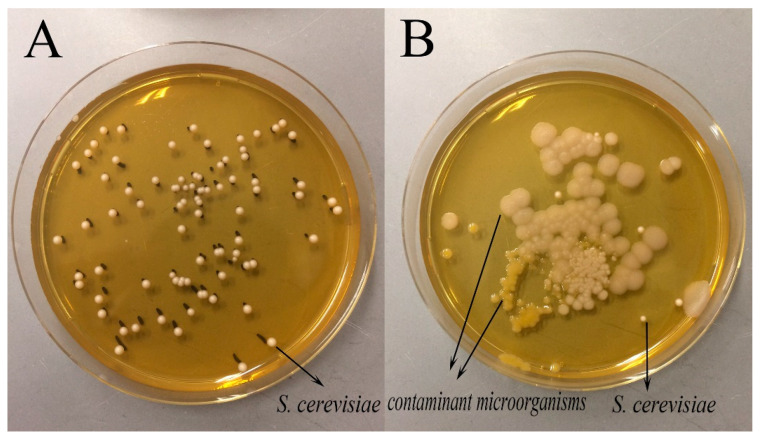
The growth of contamination on the yeast plate, (**A**) from dough fermented by *S. cerevisiae* 9Y3 and *F. sanfranciscensis* LS1, (**B**) from dough fermented by S. cerevisiae 9Y3 only.

Furthermore, it has been recently reported that *F. sanfranciscensis* could be stimulated by a secreted factor of *S. cerevisiae* [9]. However, this phenomenon was not observed in this study regarding the final bacteria concentration. Noticeably, the situation of *S. cerevisiae* was different from that of *F. sanfranciscensis*. When the dough was inoculated with *S. cerevisiae* solely, its final cell counts after 12 h were even less than the initial inoculum level. However, the amount of *S. cerevisiae* increased by one order of magnitude in the co-fermented dough. The reasons for this difference may be multi-faceted. One possible explanation is that during the dough fermentation, the inherent bacteria in the flour multiplied with a faster growth rate than *S. cerevisiae*, competing for nutrients, as shown in Figure 2, which led to the slow growth or even death of *S. cerevisiae*. However, when *F. sanfranciscensis* was present, with a speedy growth rate, it produced large amounts of acids or even possible bacteriostatic metabolites [27], which inhibited the growth of other bacteria and favored the reproduction of *S. cerevisiae* since sourdough-adapted yeasts can withstand the acidic conditions encountered during sourdough fermentation [3].

### 3.2. Volatile Compounds in the Sourdoughs

**Figure 3:**
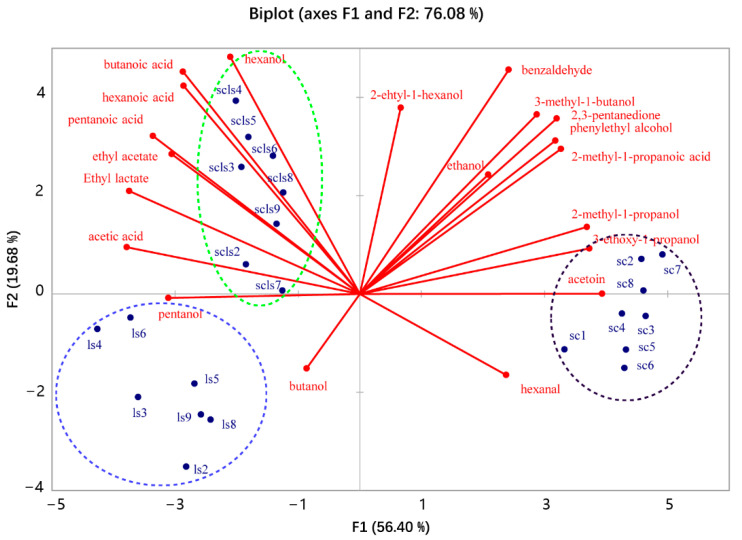
Biplot of the PCA analysis on the volatiles of sourdoughs fermented by *F. sanfranciscensis* LS1 and *S. cerevisiae* 9Y3 individually and in combination for 12 h. sc: dough fermented with *S. cerevisiae* 9Y3; ls: dough fermented with *F. sanfranciscensis* LS1. scls: dough fermented with *S. cerevisiae* 9Y3 and *F. sanfranciscensis* LS1.

A total of 20 volatile compounds were identified in the fermented doughs, including alcohols, aldehydes, ketones, acids, and esters (Table 1). A principal component analysis was carried out based on the identified compounds to compare the overall volatile profiles between the doughs. It can be found that the volatile profiles of the three fermentation groups were significantly different, separately clustered on the biplot (Figure 3), suggesting that the flavor of the co-fermented dough was distinct from those fermented by the individual strains. As shown in Figure 3 and Table 1, the co-fermented dough was characterized by higher levels of acids and esters, such as acetic acid, hexanoic acid, and ethyl acetate, which should be explained by that these compounds were produced by both the two microorganisms. The dough fermented with *S. cerevisiae* featured a higher content of alcohols and carbonyl compounds, such as 2-methyl-1-propanol and acetoin, typical metabolites of this species [28]. Among the identified compounds, 2,3-pentanedione, 2-methyl-1-propanol, acetoin, and 3-ethoxy-1-propanol were produced only by *S. cerevisiae* (Table 1). However, their concentrations were significantly decreased when *F. sanfranciscensis* was used in combination with *S. cerevisiae*, indicating that *F. sanfranciscensis* affected *S. cerevisiae* volatile production. On the other hand, ethyl lactate was the only volatile found produced by *F. sanfranciscensis* in this study. Compared with the dough fermented by *F. sanfranciscensis* alone, the content of ethyl lactate in the co-fermented dough increased slightly, not significantly (*p* > 0.05), indicating that the presence of *S. cerevisiae* did not affect the production of this ester by *F. sanfranciscensis*.

Apart from the volatiles mentioned above, the other identified compounds could be found in both of the doughs fermented by *S. cerevisiae* and *F. sanfranciscensis* and were subjected to different changes in the co-fermentation. As shown in Table 1, although both *F. sanfranciscensis* and *S. cerevisiae* could produce ethanol, the ethanol production in the co-fermented dough was slightly less, though not significantly, than the sum of the yields of the two strains alone, confirming the findings of other researchers [16], which may indicate a certain antagonism between the two strains in the utilization of carbohydrates during co-fermentation, or a detrimental effect of high acidity on ethanol production by *S. cerevisiae*. The ethyl acetate content in the co-fermented dough was approximately equal to the sum of the two fermentations, indicating that the coexistence of the two strains did not affect each other in the generation of ethyl acetate. The alcohol 3-methyl-1-butanol, one of the most important aroma compounds in bread [28], is a typical product of the Ehrlich pathway in yeast [29]. However, the content of 3-methyl-1-butanol in the co-fermented dough was significantly lower than that of the *S. cerevisiae*-fermented dough. In addition, the same was true for Phenethyl alcohol, another product of the Ehrlich pathway, indicating that *F. sanfranciscensis* had adverse effects on the production of the volatiles by *S. cerevisiae*. It is worth noting that 2-methyl-1-propanol, 3-methyl-1-butanol, and Phenethyl alcohol are the metabolites of branched-chain amino acids in yeast via the Ehrlich pathway. The significant reduction of their yields in the co-fermentation may be explained by the fact that *F. sanfranciscensis* competed with *S. cerevisiae* for amino acids during dough fermentation. As for the acids, their concentrations in the co-fermented dough were generally higher than in the single-starter fermentation but were less than the sum of individual yields of the two strains. However, the yield of butanoic acid in the co-fermented dough was significantly higher than the sum of the individual yields in single starter fermentation, indicating that the co-fermentation favored the production of butanoic acid.

### 3.3. RNA-Seq Analysis of the Sourdoughs

**Figure 4:**
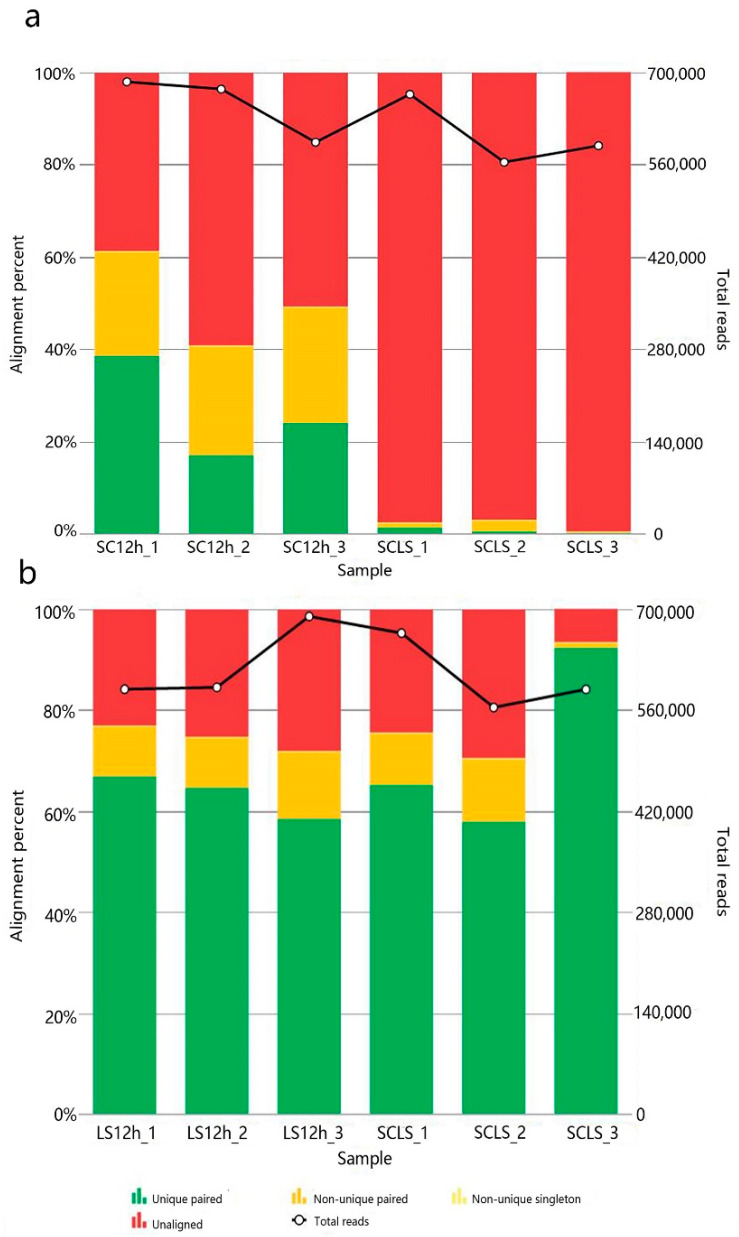
Alignment of the sequenced reads to the reference genome of *S. cerevisiae* S288C (**a**) and *F. sanfranciscensis* TMW 1.1304 (**b**). SC12h: *S. cerevisiae* 9Y3 fermentation for 12 h; LS12h: *F. sanfranciscensis* LS1 fermentation for 12 h. SCLS: co-fermentation of *S. cerevisiae* 9Y3 and *F. sanfranciscensis* LS1.

To further explore the possible interactions between *F. sanfranciscensis* and *S. cerevisiae* in the dough fermentation process, RNA sequencing (RNA-seq) analysis was performed on the co-fermented and single starter fermented doughs in the hope of peeking into the interplay of the two microorganisms concerning gene expressions. However, the amount of RNA extracted from *S. cerevisiae* in the co-fermented dough was not enough for precise analysis (Figure 4); in other words, the gene transcripts could not be effectively compared. Therefore, in this study, we focused on *F. sanfranciscensis* and investigated the gene transcriptions of *F. sanfranciscensis* with or without the absence of *S. cerevisiae*.

Total RNA concentrations extracted from the six samples (co-fermentation versus single starter fermentation, three repeats) ranged from ca.100 to 700 ng/μL, with a 260/280 ratio of around 2.0. The summary of the RNA sequencing is listed in Table S1. Total paired-end reads generated in each sample ranged from 588,303 to 689,489 with an average length of about 140 bp, and the coverage depth ranged from 135 to 233. The results showed that the transcripts of 135 genes in *F. sanfranciscensis* were significantly changed in the co-culture fermented dough compared with the single starter fermentation, with 66 genes up-regulated and 69 genes down-regulated (Table S2). The most altered genes (change fold > 5) were listed in Table 2. As shown in the table, most of the genes were associated with the transmembrane transport of amino acids, suggesting the remarkable alteration of amino acid-related metabolism in *F. sanfranciscensis* with the presence of *S. cerevisiae*.

**Figure 5:**
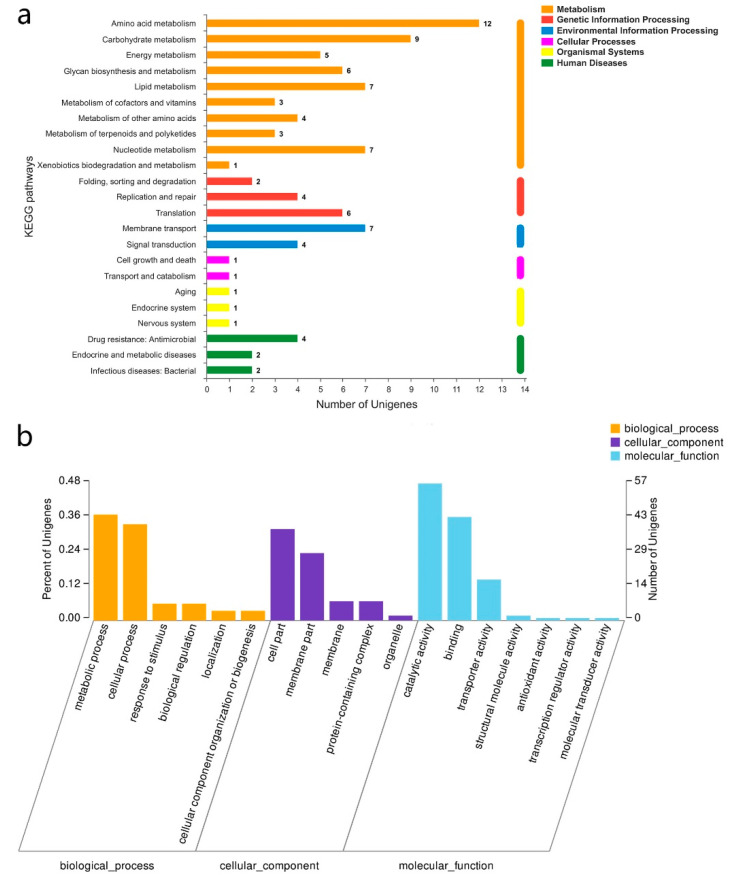
KEGG pathway annotation (**a**) and GO ontology annotation (**b**) of the differentially expressed genes in *F. sanfranciscensis* LS1 in co-fermentation.

The 135 significantly differentially expressed genes (DEGs) were subjected to KEGG and GO annotation analysis to decipher their biological functions in the metabolic activities of *F. sanfranciscensis* during the sourdough fermentation (Figure 5). The KEGG annotation showed that a large part of the DEGs was associated with metabolism. Particularly, amino acid metabolism was represented by the highest number of DEGs (12 unigenes), further demonstrating the significant influence of *S. cerevisiae* on the metabolism of amino acids in *F. sanfranciscensis* during sourdough fermentation. Following that, the carbohydrate metabolism (9 unigenes) was also markedly influenced by the presence of *S. cerevisiae*. The changes in gene expressions were in line with the previous suggestion that the importance of antagonistic and synergistic interactions between lactobacilli and yeasts were based on the metabolism of amino acids and carbohydrates and the production of carbon dioxide [15]. The GO annotation showed that the DEGs were mainly involved in 6 terms, namely, catalytic activity, binding, metabolic process, cellular process, cell part, and membrane part.

**Figure 6:**
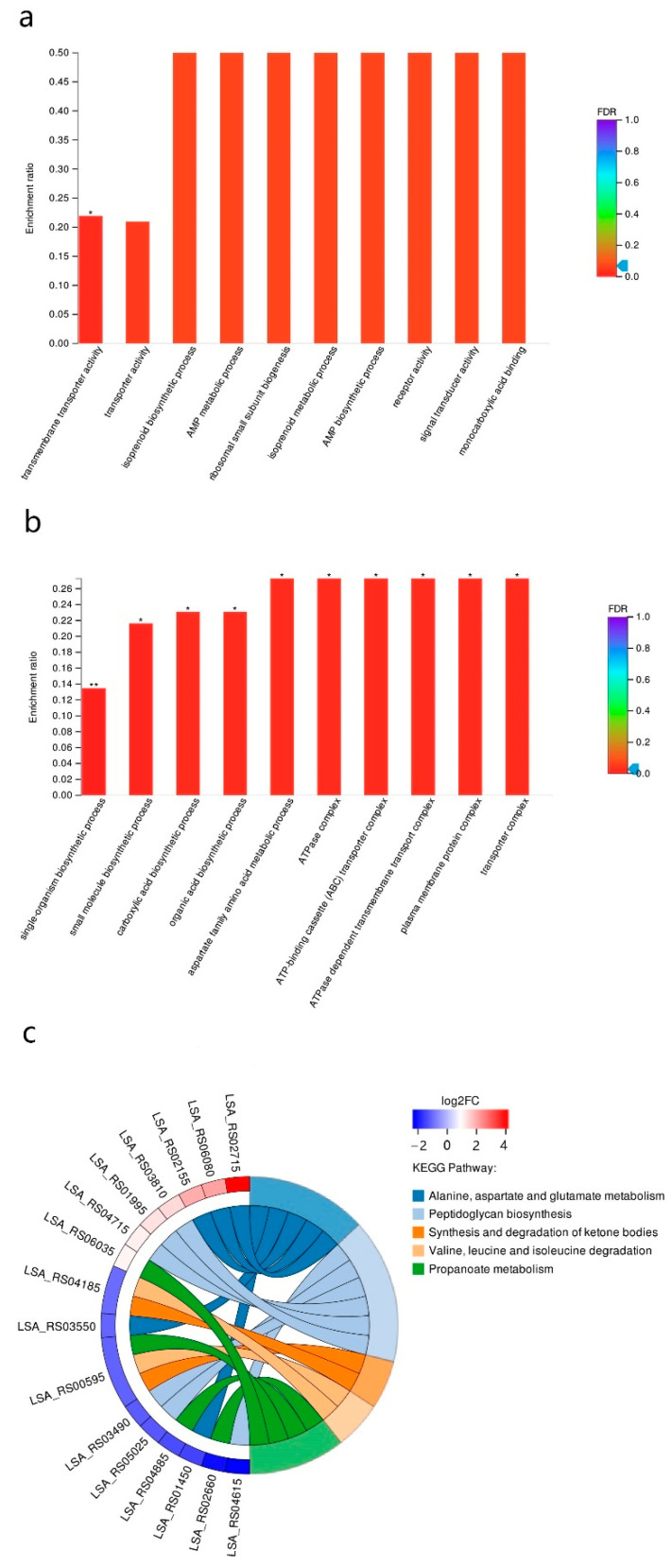
GO enrichment analysis of the total differentially expressed genes (**a**) and of the upregulated genes and the significantly enriched terms (**b**). The five most enriched KEGG pathways and the corresponding differentially expressed genes (**c**). (** *p* < 0.01, * *p* < 0.05).

Based on the function annotation, the GO and KEGG enrichment analyses were performed on the total, up-regulated, and down-regulated DEGs reported in previous studies [30,31]. As for the GO enrichment analysis, of the total DEGs, the “transmembrane transporter activity” was the only significantly enriched GO term (Figure 6a). The involved genes were mainly correlated with amino acid transmembrane transporting. However, the enrichment analysis of the up-regulated DEGs showed that dozens of GO terms were significantly enriched. The ten most enriched ones were shown in Figure 6b, mainly involving the organic acid biosynthesis process and amino acid metabolic process, and transporter complex. However, the enrichment analysis of the down-regulated DEGs showed that no GO term was significantly enriched. The enrichment revealed an overexpression of amino acid related-transmembrane transporter activity, indicating that *S. cerevisiae* greatly influenced the transmembrane transporting of amino acids in *F. sanfranciscensis* in the co-fermentation. It has been found that *S. cerevisiae* exerted significant influence, during sourdough fermentation, on the level of amino acids, generally causing a depletion of amino acids, whereas excreting specific amino acids and small peptides during growth or as a consequence of autolysis [7]. Therefore, the influence of *S. cerevisiae* on amino acid levels may account for the significant changes in gene expressions involving amino acid-related metabolic activity in *F. sanfranciscensis* in the co-fermentation. In addition, the GO enrichment analysis of the up-regulated DEGs indicated that carboxylic acid biosynthetic and organic acid biosynthetic processes in *F. sanfranciscensis* were enhanced with the presence of *S. cerevisiae*, which may explain the significant increase in butanoic acid production in the co-fermented dough.

The KEGG pathway enrichment analysis of the total, up-regulated, and down-regulated DEGs showed that no KEGG pathway was significantly overexpressed; nevertheless, some of them were markedly influenced by the presence of *S. cerevisiae* (Figure 6c), such as the pathways “Alanine, aspartate and glutamate metabolism”, “Propanoate metabolism”, “Synthesis and degradation of ketone bodies”, “Valine, leucine, and isoleucine degradation” and “Peptidoglycan biosynthesis.” It is worth noting that the transcripts of three genes involved in glutamate metabolism were significantly changed. As shown in Figure 6c, the two genes LSA_RS02715 and LSA_RS02155 encoding the enzymes glutamine-fructose-6-phosphate transaminase and type I glutamate-ammonia ligase, respectively, catalyzing the generation of D-glucosamine-6P from L-glutamate via L-glutamine were significantly upregulated, suggesting an increase in D-glucosamine-6P production, which is one of the important precursors for peptidoglycan biosynthesis [32]. Additionally, the gene LSA_RS01375, encoding N-acetyltransferase, was the most upregulated. N-acetyltransferase catalyzes the transfer of acetyl groups from acetyl-CoA to arylamines, arylhydroxylamines and arylhydrazines [33], which, in this study, was assumed to play a role in the production of N-acetyl-D-glucosamine-6-phosphate from D-glucosamine-6P, one of the most critical steps in peptidoglycan biosynthesis [34]. Furthermore, as revealed by the KEGG enrichment analysis, peptidoglycan biosynthesis was among the most enriched pathways (Figure 6c). Considering all the above, the results suggested that the peptidoglycan biosynthesis was enhanced in *F. sanfranciscensis* with the presence of *S. cerevisiae*. Since the biosynthesis of peptidoglycan is involved in binary fission during bacterial cell reproduction, the enhancement of peptidoglycan biosynthesis may imply a stimulation in the growth of *F. sanfranciscensis* by *S. cerevisiae* as recently reported [9]. However, in this study, significant differences were not observed regarding the final microbial density of *F. sanfranciscensis* in the co-fermentation, and single starter fermentation, which might explain the fact that after fermentation for 12 h, the bacterial growth in the sourdoughs both reached to stationary phase, and the *F. sanfranciscensis* strain could no longer grow below pH 3.8 [35]. Further studies may be needed to focus on the exponential phase to confirm the stimulatory effects.

## 4. Conclusions

This study revealed the mutual influence of *S. cerevisiae* and *F. sanfranciscensis* in their co-fermentation of sourdough, focusing on flavor formation and gene transcription. The presence of *S. cerevisiae* did not significantly influence the growth, acid production, and volatile generation of *F. sanfranciscensis* in sourdough fermentation; however, significant changes in gene transcription could be observed. The RNA-seq revealed that the presence of *S. cerevisiae* could alter the gene expressions implicated in amino acid metabolism and may favor the biosynthesis of peptidoglycan in *F. sanfranciscensis*. On the contrary, *F. sanfranciscensis* showed an adverse effect on the production of volatiles in *S. cerevisiae*, with some specific metabolites decreased. Nevertheless, *F. sanfranciscensis* facilitated the growth of *S. cerevisiae* in the co-fermentation, supposedly via inhibiting the nutrient competitors of *S. cerevisiae* by quickly acidifying the ecosystem. The molecular mechanism of their interactions, however, still needs future investigation.

## Figures and Tables

**Table 1:** The identified and tentatively identified volatile compounds in the fermented dough.

Chemical Group	Volatile Compounds	Calculated RI	Kovats RI of Standards	Kovats RI from Literature *	Ions Selected for Integral	Peak Areas of the Identified Volatile Compounds ^#^ (Mean Value) ×10^6^		
						LS	SC	LS + SC
Alcohols	**Ethanol**	941	938	931	31.1	87.52 ^a^	130.85 ^b^	116.09 ^b^
	**2-methyl-1-propanol**	1112	1097	1110	43.1	-	12.56 ^a^	5.03 ^b^
	butanol	1130		1146	56	0.38 ^a^	0.33 ^a^	0.32 ^a^
	**3-Methyl-1-butanol**	1217	1216	1210	55.1	0.84 ^c^	35.33 ^a^	28.65 ^b^
	**Pentanol**	1259	1259	1251	42.1	3.47 ^a^	2.21 ^b^	3.18 ^a^
	**Hexanol**	1365	1364	1352	56.1	16.1 ^a^	15.29 ^a^	18.94 ^b^
	3-ethoxy-1-propanol	1348		1359	59	-	0.45 ^b^	0.1 ^a^
	2-ethyl-1-hexanol	1459		1489	57	1.23 ^a^	1.38 ^a^	1.44 ^a^
	**Phenethyl alcohol**	1927	1921	1935	91	1.92 ^a^	17.66 ^b^	10.97 ^c^
Aldehydes	**Hexanal**	1084	1082	1080	44	2.11 ^a^	2.08 ^a^	1.24 ^a^
	**benzaldehyde**	1524	1524	1522	77	0.3 ^a^	0.67 ^b^	0.64 ^b^
Ketones	2,3-pentanedione	1065		1073	43	-	4.78 ^a^	3.46 ^b^
	acetoin	1255		1264	45.1	-	2.07 ^a^	0.33 ^b^
Acids	**Acetic acid**	1453	1447	1447	43	55.15 ^a^	2.9 ^b^	58.26 ^a^
	2-methyl-1-propanoic acid	1574		1562	43.1	0.17 ^a^	0.93 ^b^	0.59 ^c^
	Butanoic acid	1634		1623	60	0.59 ^a^	0.19 ^b^	1.08 ^c^
	**Pentanoic acid**	1744	1742	1732	60	1.01 ^a^	0.31 ^b^	1.21 ^a^
	**Hexanoic acid**	1852	1851	1842	60	4.42 ^a^	2.14 ^b^	5.43 ^a^
Esters	**Ethyl acetate**	903	892	884	61	9.66 ^a^	5.38 ^b^	14.13 ^c^
	Ethyl lactate	1351		1343	45.1	25.3 ^a^	-	28.73 ^a^

Note: The compounds in bold were identified by using authentic standards. Kovats RI from literature is the value obtained with the DB-WAX column. Kovats RI of standards was obtained under the same GC/MS conditions with samples. -: Not detected. * The Kovats RI values are from www.vcf-online.nl, accessed on 9 June 2019. ^#^ Different letters within the same row indicate a significant difference (*p* < 0.05).

**Table 2:** The most changed genes (change fold > 5) in *F. sanfranciscensis* LS1 during sourdough fermentation (co-fermentation vs. single starter).

	Gene ID	Gene Description	Change Fold	*p* Adjust
Upregulated genes				
	LSA_RS01375	N-acetyltransferase	28.40	4.03 × 10^−11^
	LSA_RS01370	ammonium transporter	22.07	4.52 × 10^−54^
	LSA_RS02715	glutamine—fructose-6-phosphate transaminase (isomerizing)	21.02	8.99 × 10^−58^
	LSA_RS00920	amino acid ABC transporter permease	20.00	3.82 × 10^−4^
	LSA_RS00925	amino acid ABC transporter ATP-binding protein	17.54	8.63 × 10^−4^
	LSA_RS00930	amino acid ABC transporter substrate-binding protein	13.22	1.48 × 10^−3^
	LSA_RS06730	amino acid ABC transporter ATP-binding protein	6.88	2.37 × 10^−35^
	LSA_RS00235	diaminopimelate decarboxylase	6.05	3.75 × 10^−5^
	LSA_RS06725	glutamine ABC transporter substrate-binding protein	5.74	8.69 × 10^−17^
	LSA_RS00340	amino acid permease	5.48	2.34 × 10^−13^
	LSA_RS03825	GatB/YqeY domain-containing protein	5.39	1.72 × 10^−11^
	LSA_RS06720	amino acid ABC transporter permease	5.02	2.55 × 10^−13^
Downregulated genes				
	LSA_RS00535	hypothetical protein	11.11	0.03
	LSA_RS00005	chromosomal replication initiator protein DnaA	6.90	6.35 × 10^−18^
	LSA_RS01215	alpha/beta hydrolase	6.58	6.20 × 10^−11^
	LSA_RS05425	DUF1304 domain-containing protein	5.78	8.57 × 10^−10^
	LSA_RS04335	aminoglycoside phosphotransferase	5.52	8.13 × 10^−27^
	LSA_RS04615	D-alanine—D-alanine ligase A	5.49	1.51 × 10^−23^
	LSA_RS06125	ABC transporter permease	5.376344	1.98 × 10^−3^
	LSA_RS04340	amino acid permease	5.128205	8.13 × 10^−27^
	LSA_RS00545	ABC transporter ATP-binding protein	5.05	6.84 × 10^−3^
	LSA_RS01530	nitronate monooxygenase	5.00	1.68 × 10^−15^

## Data Availability

Data is contained within the article or supplementary material.

## Supplementary Materials

The following are available online at https://www.mdpi.com/article/10.3390/foods11152373/s1, Table S1: Run summary on an Illumina MiSeq platform, Table S2. The significantly changed genes in the sourdoughs (co-fermentation vs. single starter).
