# Supplementary material for: The Mutual Influence of Predominant Microbes in Sourdough Fermentation: Focusing on Flavor Formation and Gene Transcription
Source: Foods. 2022 Aug 8;11(15):2373. doi: 10.3390/foods11152373 (PMC9367918; doi:10.3390/foods11152373)
Supplement: Supplementary file 1 [file foods-11-02373-s001.zip › foods-1830713-supplementary.pdf]

Table S1. Run summary on an Illumina MiSeq platform

Sample name	Total reads	Total alignments	Aligned	Unique paired	Non-unique paired	Coverage	Average coverage depth	Aver-age length	GC content
SCLS1	666,319	1,711,368	75.59%	65.17%	10.41%	84.26%	204.65	139.00	42.95%
SCLS2	563,182	1,522,988	70.55%	57.88%	12.67%	83.32%	184.60	139.54	43.80%
SCLS3	588,303	1,139,608	93.55%	92.39%	1.16%	84.91%	135.98	139.81	38.46%
LS1	588,507	1,499,598	76.88%	66.94%	9.94%	84.68%	179.97	140.46	42.83%
LS2	591,061	1,493,230	74.70%	64.61%	10.09%	83.68%	181.40	140.41	43.30%
LS3	689,489	1,971,612	71.98%	58.52%	13.46%	84.84%	233.92	138.93	43.95%

Table S2. The significantly changed genes in the sourdoughs (co-fermentation vs. single starter)

Gene_id	Gene description	FC(SCLS/LS)	Pvalue	Padjust
LSA_RS01375	N-acetyltransferase	28.397	1E-12	4.03E-11
LSA_RS01370	ammonium transporter	22.073	8.65E-57	4.52E-54
LSA_RS02715	glutamine--fructose-6-phosphate transaminase (isomerizing)	21.018	8.61E-61	8.99E-58
LSA_RS00920	amino acid ABC transporter permease	19.997	3.87E-05	0.000382
LSA_RS00925	amino acid ABC transporter ATP-binding protein	17.537	9.91E-05	0.000863
LSA_RS00930	amino acid ABC transporter substrate-binding protein	13.216	0.000192	0.001478
LSA_RS06730	amino acid ABC transporter ATP-binding protein	6.879	6.81E-38	2.37E-35
LSA_RS00235	diaminopimelate decarboxylase	6.046	2.8E-06	3.75E-05
LSA_RS06725	glutamine ABC transporter substrate-binding protein	5.736	9.14E-19	8.69E-17
LSA_RS00340	amino acid permease	5.483	4.03E-15	2.34E-13
LSA_RS03825	GatB/YqeY domain-containing protein	5.392	3.79E-13	1.72E-11
LSA_RS06720	amino acid ABC transporter permease	5.018	4.64E-15	2.55E-13
LSA_RS06080	adenylosuccinate synthase	4.537	2.53E-24	3.78E-22
LSA_RS02150	MerR family DNA-binding transcriptional regulator	4.52	1.18E-16	8.8E-15
LSA_RS01190	Cof-type HAD-IIB family hydrolase	4.248	1.56E-15	9.6E-14
LSA_RS02155	type I glutamate--ammonia ligase	4.167	3.99E-18	3.48E-16
LSA_RS03165	5-formyltetrahydrofolate cyclo-ligase	4.003	0.004904	0.021003
LSA_RS06715	amino acid ABC transporter permease	3.996	2.22E-12	8.02E-11
LSA_RS00240	2%2C3%2C4%2C5-tetrahydropyridine-2%2C6-dicarboxylate N-acetyltransferase	3.79	1E-08	2.43E-07
LSA_RS00210	DUF536 domain-containing protein	3.637	0.000285	0.00196
LSA_RS04000	hypothetical protein	3.599	1.33E-06	1.88E-05
LSA_RS03770	Nif3-like dinuclear metal center hexameric protein	3.494	3.73E-05	0.000375
LSA_RS03775	SAM-dependent methyltransferase	3.395	0.001456	0.007495
LSA_RS04470	DNA polymerase IV	3.333	0.000153	0.001251
LSA_RS02720	hypothetical protein	3.166	0.000195	0.00148
LSA_RS04010	transcription termination/antitermination protein NusA	3.115	7.39E-14	3.68E-12
LSA_RS04005	DUF448 domain-containing protein	3.09	3.11E-08	6.92E-07
LSA_RS03650	ribonuclease HII	3.081	0.000169	0.001349
LSA_RS01990	CTP synthase	2.956	5.56E-13	2.42E-11
LSA_RS04015	ribosome maturation factor RimP	2.917	2.21E-07	3.91E-06
LSA_RS02760	DUF805 domain-containing protein	2.842	8.72E-05	0.000779
LSA_RS02490	ABC transporter ATP-binding protein	2.828	5.22E-08	1.11E-06
LSA_RS03810	diacylglycerol kinase	2.823	5.52E-07	8.88E-06
LSA_RS02525	PDZ domain-containing protein	2.796	4.06E-06	4.93E-05

LSA_RS03985	tRNA pseudouridine(55) synthase TruB	2.788	0.007411	0.029579
LSA_RS02470	co-chaperone GroES	2.78	8.19E-10	2.19E-08
LSA_RS06065	3-hydroxyacyl-[acyl-carrier-protein] dehydratase FabZ	2.522	0.004671	0.020424
LSA_RS03980	bifunctional riboflavin kinase/FAD synthetase	2.51	0.00047	0.002921
LSA_RS01995	UDP-N-acetylglucosamine 1-carboxyvinyltrans-ferase	2.498	2.63E-11	8.57E-10
LSA_RS00245	N-acetyldiaminopimelate deacetylase	2.488	1.02E-06	1.52E-05
LSA_RS02520	peptide chain release factor 2	2.463	0.000214	0.001577
LSA_RS04155	MFS transporter	2.431	0.001582	0.007986
LSA_RS06780	tRNA uridine-5-carboxymethylaminomethyl(34) synthesis GTPase MnmE	2.428	3E-07	5.06E-06
LSA_RS03820	PhoH family protein	2.409	4.66E-07	7.61E-06
LSA_RS02475	chaperonin GroEL	2.383	8.65E-07	1.35E-05
LSA_RS02010	MFS transporter	2.374	0.000497	0.003057
LSA_RS03510	class I SAM-dependent RNA methyltransferase	2.312	9.65E-08	1.9E-06
LSA_RS03800	DNA repair protein RecO	2.295	2.86E-06	3.75E-05
LSA_RS03805	GTPase Era	2.261	2.55E-09	6.5E-08
LSA_RS03425	mevalonate kinase	2.231	0.001575	0.007986
LSA_RS04715	DUF1727 domain-containing protein	2.182	1.11E-07	2.16E-06
LSA_RS01185	transcriptional regulator Spx	2.178	1.5E-07	2.7E-06
LSA_RS06035	acetyl-CoA carboxylase biotin carboxyl carrier protein subunit	2.172	0.000635	0.003814
LSA_RS03815	rRNA maturation RNase YbeY	2.167	1.08E-05	0.000121
LSA_RS04420	DUF2507 domain-containing protein	2.167	0.005268	0.022021
LSA_RS02535	HPr kinase/phosphorylase	2.141	2.66E-07	4.56E-06
LSA_RS06735	cardiolipin synthase	2.106	2.52E-05	0.000263
LSA_RS00215	L%2CD-transpeptidase	2.102	9.03E-07	1.39E-05
LSA_RS02100	Hsp33 family molecular chaperone HslO	2.102	2.32E-05	0.000245
LSA_RS03515	FAD-binding oxidoreductase	2.094	5.2E-06	6.25E-05
LSA_RS02620	triose-phosphate isomerase	2.092	0.000163	0.001308
LSA_RS06460	GMP reductase	2.085	0.000141	0.001166
LSA_RS06025	acetyl-CoA carboxylase biotin carboxylase subu-nit	2.04	3.11E-06	4.01E-05
LSA_RS02000	type B 50S ribosomal protein L31	2.014	3.72E-06	4.58E-05
LSA_RS00195	MFS transporter	2.006	0.001442	0.007481
LSA_RS06220	stage V sporulation protein M	2.002	1.84E-05	0.000196
LSA_RS03860	N-acetylmuramoyl-L-alanine amidase	0.498	0.00342	0.015471
LSA_RS00020	DNA replication and repair protein RecF	0.494	6.73E-05	0.000634
LSA_RS04185	hydroxymethylglutaryl-CoA synthase	0.493	2.8E-08	6.38E-07
LSA_RS06425	LysM peptidoglycan-binding domain-containing protein	0.491	7.76E-10	2.13E-08
LSA_RS01115	divalent metal cation transporter	0.49	2.56E-06	3.47E-05
LSA_RS00695	sodium:proton antiporter	0.49	0.00022	0.001594

LSA_RS03445	polyprenyl synthetase family protein	0.487	8.8E-05	0.000779
LSA_RS02455	LemA family protein	0.484	0.000248	0.001772
LSA_RS04970	isoleucine--tRNA ligase	0.482	8.55E-08	1.72E-06
LSA_RS01915	DUF72 domain-containing protein	0.473	8.72E-06	0.000102
LSA_RS04875	translational GTPase TypA	0.466	8.56E-12	2.89E-10
LSA_RS01580	RluA family pseudouridine synthase	0.462	0.003096	0.014126
LSA_RS06345	flippase	0.459	1.25E-07	2.34E-06
LSA_RS01825	DUF4811 domain-containing protein	0.458	0.004981	0.021245
LSA_RS03550	ATP-grasp domain-containing protein	0.457	3.7E-06	4.58E-05
LSA_RS04575	cysteine desulfurase	0.456	1.23E-05	0.000135
LSA_RS04745	ribonuclease Z	0.452	0.000803	0.004634
LSA_RS03555	DUF814 domain-containing protein	0.449	2.54E-06	3.47E-05
LSA_RS03405	23S rRNA methyltransferase	0.445	0.000134	0.001123
LSA_RS01300	protein-tyrosine-phosphatase	0.445	0.000444	0.002814
LSA_RS00595	acetyl-CoA C-acyltransferase	0.444	2.74E-05	0.000284
LSA_RS01225	LTA synthase family protein	0.434	1.2E-07	2.28E-06
LSA_RS04730	adenine phosphoribosyltransferase	0.432	0.000618	0.003735
LSA_RS04980	RNA-binding protein	0.43	7.78E-08	1.59E-06
LSA_RS03490	penicillin-binding protein 1A	0.424	1.02E-09	2.67E-08
LSA_RS05940	universal stress protein	0.423	1.38E-05	0.00015
LSA_RS04095	transcriptional repressor LexA	0.42	0.000117	0.000999
LSA_RS00125	sulfite exporter TauE/SafE family protein	0.414	0.000102	0.00088
LSA_RS05025	PASTA domain-containing protein	0.413	6.92E-09	1.72E-07
LSA_RS05935	3-methyladenine DNA glycosylase	0.41	3.72E-06	4.58E-05
LSA_RS05565	energy-coupling factor transporter ATPase	0.409	0.011175	0.041413
LSA_RS02670	GNAT family N-acetyltransferase	0.404	0.000176	0.001384
LSA_RS06660	NAD(P)-dependent oxidoreductase	0.397	9.44E-06	0.00011
LSA_RS06270	methylated-DNA--[protein]-cysteine S-methyl-transferase	0.384	0.000338	0.002231
LSA_RS02665	tRNA (adenosine(37)-N6)-threonylcar-bamoyltransferase complex ATPase subunit type 1 TsaE	0.379	4.38E-05	0.00042
LSA_RS04795	30S ribosomal protein S15	0.373	6.58E-11	1.96E-09
LSA_RS02305	bifunctional lysylphosphatidylglycerol flip-pase/synthetase MprF	0.372	4.6E-08	1E-06
LSA_RS01455	hypothetical protein	0.372	3.29E-06	4.2E-05
LSA_RS01820	MFS transporter	0.361	3.3E-11	1.01E-09
LSA_RS03265	guanylate kinase	0.361	2.81E-08	6.38E-07
LSA_RS03310	50S ribosomal protein L28	0.354	1.25E-06	1.8E-05
LSA_RS01480	APC family permease	0.341	2.22E-12	8.02E-11
LSA_RS01450	asparaginase	0.338	1.81E-08	4.3E-07
LSA_RS06265	glutamate/gamma-aminobutyrate family trans-porter YjeM	0.336	5.56E-12	1.94E-10
LSA_RS04885	lactate dehydrogenase	0.335	0.00018	0.001391

LSA_RS00010	DNA polymerase III subunit beta	0.305	3.92E-16	2.73E-14
LSA_RS06130	hypothetical protein	0.301	1.31E-22	1.71E-20
LSA_RS02735	asparagine--tRNA ligase	0.301	7.09E-21	8.23E-19
LSA_RS00285	amino acid permease	0.299	1.66E-13	7.89E-12
LSA_RS01400	alpha/beta hydrolase	0.29	0.000487	0.003011
LSA_RS06470	amino acid permease	0.288	5.84E-07	9.25E-06
LSA_RS03000	DNA translocase FtsK	0.279	7.53E-13	3.15E-11
LSA_RS01535	amino acid permease	0.256	1.22E-10	3.54E-09
LSA_RS00430	hypothetical protein	0.254	3.05E-07	5.06E-06
LSA_RS00290	ASCH domain-containing protein	0.253	7.76E-08	1.59E-06
LSA_RS01620	threonine/serine exporter	0.247	0.000283	0.001959
LSA_RS01295	tyrosine--tRNA ligase	0.241	2.18E-10	6.17E-09
LSA_RS00830	IS256 family transposase	0.218	2.41E-14	1.26E-12
LSA_RS02660	phosphate acetyltransferase	0.209	9.22E-16	6.02E-14
LSA_RS01530	nitronate monooxygenase	0.2	2.09E-17	1.68E-15
LSA_RS00545	ABC transporter ATP-binding protein	0.198	0.001283	0.006839
LSA_RS04340	amino acid permease	0.195	3.89E-29	8.13E-27
LSA_RS06125	ABC transporter permease	0.186	0.000294	0.001984
LSA_RS04615	D-alanine--D-alanine ligase A	0.182	8.66E-26	1.51E-23
LSA_RS04335	aminoglycoside phosphotransferase	0.181	3.82E-29	8.13E-27
LSA_RS05425	DUF1304 domain-containing protein	0.173	2.71E-11	8.57E-10
LSA_RS01215	alpha/beta hydrolase	0.152	1.6E-12	6.2E-11
LSA_RS00005	chromosomal replication initiator protein DnaA	0.145	6.08E-20	6.35E-18
LSA_RS00535	hypothetical protein	0.09	0.006301	0.025622
